# NKT Cells as an Ideal Anti-Tumor Immunotherapeutic

**DOI:** 10.3389/fimmu.2013.00409

**Published:** 2013-12-02

**Authors:** Shin-ichiro Fujii, Kanako Shimizu, Yoshitaka Okamoto, Naoki Kunii, Toshinori Nakayama, Shinichiro Motohashi, Masaru Taniguchi

**Affiliations:** ^1^Laboratory for Immunotherapy, RCAI, RIKEN, Center for Integrative Medical Sciences (IMS-RCAI), Yokohama, Japan; ^2^Department of Otorhinolaryngology, Graduate School of Medicine, Chiba University, Chiba, Japan; ^3^Department of Immunology, Graduate School of Medicine, Chiba University, Chiba, Japan; ^4^Department of Medical Immunology, Graduate School of Medicine, Chiba University, Chiba, Japan; ^5^Laboratory of Immune Regulation, RCAI, RIKEN, Center for Integrative Medical Sciences (IMS-RCAI), Yokohama, Japan

**Keywords:** NKT cells, adjuvant effects, clinical trial, induced pluripotent stem cells, artificial adjuvant vector cells

## Abstract

Human natural killer T (NKT) cells are characterized by their expression of an invariant T cell antigen receptor α chain variable region encoded by a Vα24Jα18 rearrangement. These NKT cells recognize α-galactosylceramide (α-GalCer) in conjunction with the MHC class I-like CD1d molecule and bridge the innate and acquired immune systems to mediate efficient and augmented immune responses. A prime example of one such function is adjuvant activity: NKT cells augment anti-tumor responses because they can rapidly produce large amounts of IFN-γ, which acts on NK cells to eliminate MHC negative tumors and also on CD8 cytotoxic T cells to kill MHC positive tumors. Thus, upon administration of α-GalCer-pulsed DCs, both MHC negative and positive tumor cells can be effectively eliminated, resulting in complete tumor eradication without tumor recurrence. Clinical trials have been completed in a cohort of 17 patients with advanced non-small cell lung cancers and 10 cases of head and neck tumors. Sixty percent of advanced lung cancer patients with high IFN-γ production had significantly prolonged median survival times of 29.3 months with only the primary treatment. In the case of head and neck tumors, 10 patients who completed the trial all had stable disease or partial responses 5 weeks after the combination therapy of α-GalCer-DCs and activated NKT cells. We now focus on two potential powerful treatment options for the future. One is to establish artificial adjuvant vector cells containing tumor mRNA and α-GalCer/CD1d. This stimulates host NKT cells followed by DC maturation and NK cell activation but also induces tumor-specific long-term memory CD8 killer T cell responses, suppressing tumor metastasis even 1 year after the initial single injection. The other approach is to establish induced pluripotent stem (iPS) cells that can generate unlimited numbers of NKT cells with adjuvant activity. Such iPS-derived NKT cells produce IFN-γ *in vitro* and *in vivo* upon stimulation with α-GalCer/DCs, and mediated adjuvant effects, suppressing tumor growth *in vivo*.

## Discovery of NKT Cells Expressing an Invariant Vα14Jα18 Antigen Receptor

Natural killer T (NKT) cells are characterized by the expression of an invariant antigen receptor encoded by Vα14Jα18 in mice and Vα24Jα18 in humans (1–3). The murine invariant Vα14Jα18 NKT cell antigen receptor was identified by cloning of cDNAs encoding T cell antigen receptor (TCR) from 13 independently established hybridomas with regulatory functions (4, 5). Surprisingly at that time, Southern blot analysis of TCR usage by these 13 hybridomas had the same DNA restriction fragment length polymorphism (RFLP) patterns, even when three different enzymes, *Eco*RI, *Bam*HI, and *Hin*dIII were used. Because of this unusual homogeneous DNA restriction pattern, the TCR cDNAs were cloned and could be classified into four types at the nucleotide level, all of which were composed of Vα14 and Jα18 with a 1-nt N region. The N region was different in each clone, a C, A, T, or G nucleotide. However, any nucleotide addition in the N region at this position becomes invariant at the amino acid level, because this N region is the third base of a glycine codon (5).

By RNase protection assays using antisense Vα14Jα18 of C57BL/6 (B6) origin as a probe, we detected a single 630 bp band in B6, a single 400 bp band in BALB/c, and 630/400 double bands in DBA/2 mice. Quite remarkably, this band(s) represented 2–4% in the total TCRα expression in these mice (6). The theoretical expression frequency of any one particular TCRα is calculated to be 1/10^6^, because the total TCRα chain repertoire is around 10^8^ and there are 100 Vα segments in the TCRα loci. Therefore, the Vα14Jα18 expression frequency detected in unprimed mice was more than 10^4^ times higher than expected, suggesting that Vα14^+^ NKT cells are clonally expanded under physiological conditions, likely do to their intrinsic autoreactivity. Another interesting finding was that the invariant Vα14Jα18 receptor is used only by NKT cells and not by conventional αβ T cells. This was shown conclusively when the invariant Vα14Jα18 together with TCRVβ8.2 was introduced into RAG-knockout (KO) mice; only NKT cells and not conventional αβ T cells or NK cells developed (7). These and other studies confirmed that expression of Vα14Jα18 in mice and Vα24Jα18 in human is a unique NKT cell signature.

## Discovery of the NKT Cell Ligand

The ligand for NKT cells was identified as α-galactosylceramide (α-GalCer), which is presented by the MHC class I-like CD1d molecule. However, unlike MHC class I molecule with polymorphic in nature, CD1d is monomorphic among species, indicating that α-GalCer can be used in any potential NKT cell therapy for all humans. The glycolipid nature of the NKT cell ligand was suggested by experiments using mice lacking the transporter associated with antigen processing (TAP), which is essential for translocation of cytoplasmic peptides generated by the ubiquitin-proteasome proteolytic pathway into the endoplasmic reticulum (ER) to make a stable complex with MHC class I molecules. The MHC peptide complex is required to select CD8 T cells, therefore, in TAP-KO mice, CD8 T cells are not generated. However, by RNase protection assays using the invariant Vα14Jα18 as a probe, we could detect significant levels of protected bands in TAP-KO mice but not in β2M-KO mice, suggesting that the ligand is not a peptide, but likely to be a glycolipid in conjunction with a β2M-associated MHC-like molecule (8). The MHC-like molecule turned out to be CD1d, which has two large hydrophobic pockets, A′ and F′, that can bind the two long fatty acid chains of the ceramide portion of α-GalCer (9). Therefore, we screened various synthetic glycolipids and found the essential structure-function relationships critical for the NKT cell recognition, such as: (1) α-linkage between the sugar moiety and the ceramide portion of α-GalCer but not β-GalCer, (2) a 2′-OH configuration on the sugar moiety different from α-ManCer, and (3) a 3′-OH on the sphingosine of α-GalCer (10).

Furthermore, by using alanine substitution to mutagenize CD1d, we also identified important amino acids on CD1d, such as Ser76, Arg79, Asp80, Glu83, and Gln153, for activation of NKT cells in mice (11). In 2007, Borg et al. succeeded in crystallizing the triple complex of α-GalCer/human Vα24Jα18/TCRVβ11/human CD1d (12). Interestingly, the Vα24Jα18 chain docks in parallel with the CD1d cleft without any direct contribution of the TCRβ chain to ligand binding. This configuration is quite different from the mode of ligand recognition by the TCRβ chain of conventional αβ T cells, in which only the TCRβ but not the TCRα chain recognizes the MHC bound peptide in a diagonal position.

Analysis of the structure also revealed that the first four amino acids (Asp94, Arg95, Gly96, and Ser97) of Jα18, which are conserved in mouse and human, are essential for binding with both CD1d and α-GalCer. The Jα18Asp94 binds with CD1dArg79, Jα18Arg95 with CD1dArg79/Ser76/Asp80 and the 3′-OH on the sphingosine, Jα18Gly96 with the 2′-OH on galactose, and Jα18Ser97 with CD1dGln150. Interestingly, the CD1d amino acid, Glu83, defined as important in functional assays with CD1d mutants, is important for binding with the TCRβ chain to make a stable complex with CD1d but has no direct contribution to the ligand binding itself. Moreover, the CD1d amino acids (Ser76, Arg79, and Asp80) important for binding with either α-GalCer or Jα18 are also well conserved among species such as mouse, rat, sheep, and human (10, 13–15). Thus, α-GalCer, identified as an NKT cell ligand in mice can also be used to activate human NKT cells.

## NKT Cell-Mediated Adjuvant Effects on Innate and Adaptive Immunity Against Cancer

**Figure 1:**
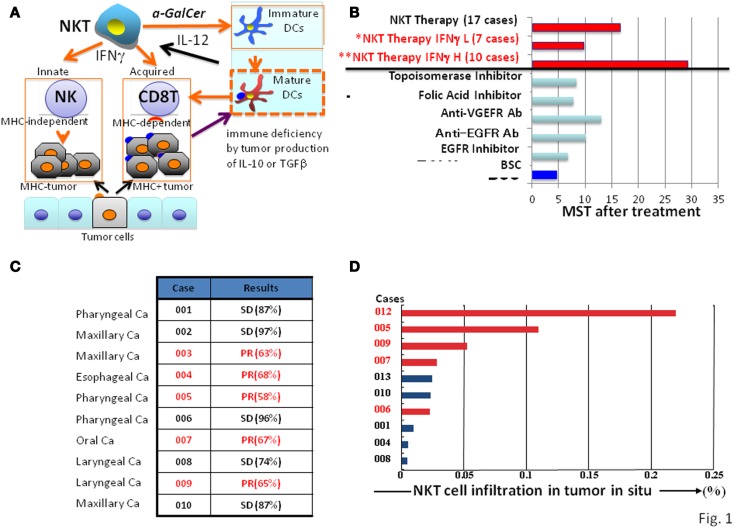
**Natural killer T cell-mediated adjuvant effects on anti-tumor protective responses and clinical trial outcomes**. **(A)** Mechanisms of NKT cell-targeted adjuvant cell therapy: upon NKT cell activation in patients by α-GalCer/DCs, immature DC become mature, and both MHC positive and negative tumor cells will be killed by CD8 killer T cells and NK cells, respectively. **(B)** Clinical trials of NKT cell-targeted adjuvant cell therapy on advanced non-small lung cancer: 60% of patients (**) showed significant prolonged median survival time of 29.3 months compared with best supportive care group with a MST of 4.6 months. The response to NKT cell therapy correlated with clinical efficacy (median survival time) and IFN-γ levels; patients with high (**H) levels responded significantly better than those with low (*L) levels. **(C)** Clinical trials of NKT cell-targeted adjuvant cell therapy for head and neck tumors: all 10 cases treated with the combination therapy of α-GalCer/DCs and activated NKT cells showed significant clinical efficacy (SD or PR). **(D)** Correlation between clinical efficacy (PR in red, SD in black) of head and neck tumors and NKT cell numbers in the tumor *in situ*.

In general, tumor cells do not contain any adjuvant materials, so that it is difficult to induce proliferation of specific T cell clones to mount anti-tumor responses in patients. On this particular point, α-GalCer overcomes these problems by its intrinsic adjuvant activity, inducing clonal expansion of tumor-specific T cell cells as well as activating various innate cell types (16). In the initial anti-tumor response after stimulation with α-GalCer/DCs, NKT cells immediately produce large amounts of IFN-γ, which acts on DCs, NK cells, and neutrophils in the innate immune system to eliminate MHC negative tumor target cells and, at the same, also on CD8 cytotoxic T cells and CD4 Th1 cells to kill MHC positive tumor cells, resulting in tumor eradication (Figure 1) (1, 17, 18). Therefore, NKT cell-targeted therapy is expected to overcome the major problem of current anti-cancer immunotherapies – recurrent tumors – due to their targeting of only one type of effector cell (10, 19, 20). For example, in the immunotherapy using tumor peptide CTL or antibodies against PD-1 or CTLA4, the target is the CD8 killer T cell, which kills MHC positive but not negative tumor cells, resulting in tumor recurrence (21). Similarly, in the artificial cells recently developed by the forced expression of Rae1/H60 (NKG2D-L), Mult-1 (NKG2D-L), or CD70 (TNF-L), the target cells are NK cells, which will eliminate MHC negative, but not MHC positive tumor cells (22).

Tumors in general contain both MHC positive and negative cells. Therefore, for an optimal therapy, both MHC types of tumor cells should be eliminated simultaneously by activating both innate and adaptive immune responses (Figure 1A). Since only NKT cells, but not other immune cells, activate NK and CD8 killer T cells at the same time, thus eliminating both MHC positive and negative tumor cells, the NKT cell-targeted therapy is a promising strategy for cancer treatment (Figures 1B,C).

## NKT Cell-Mediated Adjuvant Effects on DC Maturation

Another important NKT cell function is their ability to interact with immature DCs in the presence of α-GalCer to induce DC maturation (17). Therefore, NKT cell-targeted therapy is also useful for advanced cancer patients, who often suffer from severe immunodeficiency. DCs in these advanced cancer patients are usually immature because of the presence of immune suppressive cytokines, such as IL-10 or TGFβ, produced by tumor cells (Figure 1A) (23). The immature DCs are able to capture tumor antigens, but unable to activate specific T cells. However, immature DCs presenting α-GalCer are activated by NKT cells through CD40-CD40L interactions to produce IFN-γ, which induce full DC maturation (24). This leads to a robust interleukin (IL)-12 response to further activate NKT cells, followed by activation of CD8T cells and NK cells (17, 24).

**Figure 2:**
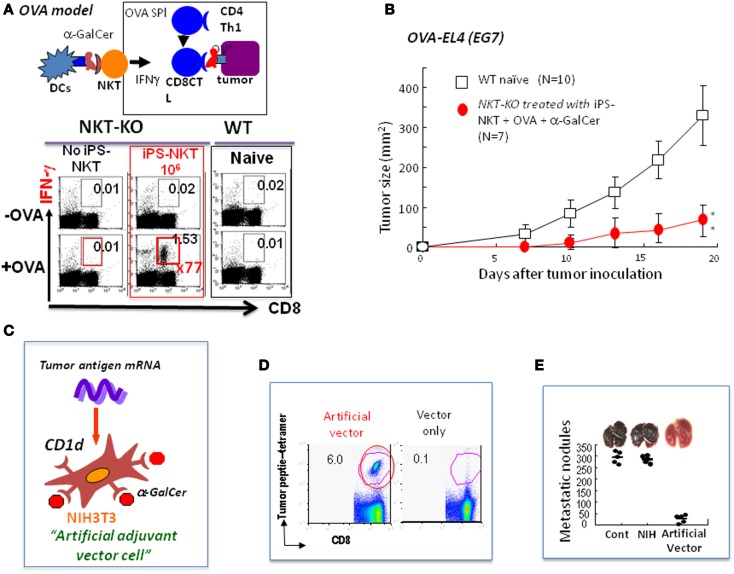
**Future directions for NKT cell-mediated cancer therapy**. **(A)** Experimental model using OVA as an artificial tumor antigen to demonstrate NKT cell-mediated adjuvant activity (OVA model): the NKT-KO mice that had received iPS-derived NKT cells were immunized with OVA-loaded TAP-deficient spleen cells permeabilized by osmotic shock. A week later, the CD8 killer T cells in these immunized mice were analyzed for IFN-γ production after restimulation with OVA antigen *in vitro*. A significant increase in the number of antigen-specific IFN-γ producing CD8 killer T cells was detected in mice transferred with iPS-derived NKT cells. **(B)** Inhibition of the growth of OVA-expressing EL4 (EG7) tumor cells by NKT cell-mediated adjuvant therapy using iPS-derived NKT cells *in vivo* in the OVA model. A significant suppression of tumor growth *in vivo* was detected. **(C)** Generation of allogeneic artificial adjuvant vector cells. Artificial adjuvant vector cells were loaded with α-GalCer/CD1d and transfected with tumor mRNA. **(D)** Detection of long-term memory antigen-specific CD8 killer T cells even 1 year after a single injection of artificial adjuvant vector cells. Antigen-specific CD8 T cell responses in mice immunized with artificial adjuvant vector cells were analyzed using tetramer staining 1 year later. OVA was used in these experiments. **(E)** Suppression of melanoma lung metastasis after treatment with artificial adjuvant vector cells. Mice were intravenously injected with B16 melanoma cells to induce lung metastasis and, then 3 h later, intravenously with artificial adjuvant vector cells without tumor mRNA. The formation of metastatic nodules analyzed 2 weeks after melanoma cell injection was significantly suppressed according to the mechanisms of the activation of both NKT and NK cells but not that of CD8 killer T cells induced by artificial adjuvant vector cells carrying only α-GalCer/CD1d without tumor mRNA.

The DC maturation by activated NKT cells is a prominent strategy for the enhancement of protective innate and acquired immune responses. To investigate the mechanisms of bystander potential of α-GalCer-activated NKT cells, an experimental system using immunization with OVA-loaded TAP-deficient spleen cells loaded with OVA after permeabilization by osmotic shock was developed. In this system, OVA was used as an artificial tumor antigen to induce OVA-specific CD8 T cells to kill OVA-bearing tumor cells. Only after α-GalCer administration, IFN-γ production by NK and CD8T cells was observed (see Figure 2A). Under these conditions, the clonal expansion of OVA-specific CD8 T cells and strong anti-tumor responses develop in the mice, and the response requires co-administration of α-GalCer (17).

## Clinical Trial of NKT Cell-Targeted Therapy for Advanced Lung Cancer and Head and Neck Tumors

For effective NKT cell activation, α-GalCer/DC has distinct advantages to induce significant expansion of NKT cells and to inhibit *in vivo* tumor growth in a mouse model of metastatic lung cancer and liver metastasis in melanoma (25, 26). In a preclinical study, we used mouse melanoma cells, which were injected into the spleen to induce liver metastasis. Treatment of tumor-bearing mice by intravenous administration of α-GalCer/DCs (3 × 10^6^) resulted in complete eradication of the liver metastasis within 7 days after treatment (27).

Based on the dramatic effects of α-GalCer/DCs in the preclinical studies, a clinical trial of NKT cell-targeted immunotherapy was conducted at Chiba University hospital in patients with advanced non-small cell lung cancer to evaluate the safety, feasibility, immunological responses, and clinical outcomes (28). Seventeen patients with advanced or recurrent non-small cell lung cancer refractory to the standard treatments, including surgery, chemotherapy, and radiation therapy, completed the protocol. The patient’s peripheral blood mononuclear cells (PBMCs) obtained by apheresis were cultured with GMP grade GM-CSF and IL-2 for 7 days and then pulsed with α-GalCer (29). The α-GalCer-pulsed PBMCs were then intravenously administered (1 × 10^9^ cells/m^2^/injection) back into autologous patients twice with a 1-week interval followed by two courses with a 1-month interval between the second and third administration.

In the 17 patients who completed the protocol of a phase IIa clinical trial, the treatment was well-tolerated, and no severe adverse events related to the cell therapy were observed (28, 30). To monitor IFN-γ production by NKT cells from the patients, an enzyme-linked immunospot (ELISPOT) assay was performed (31). The results demonstrated that a significant increase in the number of IFN-γ-producing PBMCs was detected in 10 out of 17 patients, which was correlated with a significantly prolonged median survival time (MST; 29.3 months) in comparison with the group with no increase compared to the pretreatment status in IFN-γ-producing cells (MST of 9.7 months) (Figure 1B) (32). The α-GalCer-reactive IFN-γ spot forming cells appeared to include both NKT cells and NK cells (31, 33), consistent with the notion that α-GalCer-activated NKT cells subsequently stimulate NK cells to produce IFN-γ (34, 35). We also investigated NKT cell infiltration in the surgically resected tumor samples and found a significant increase (25- to 60-fold) in the number of NKT cells in the tumor *in situ* (36). Because of the clinical correlation between increased IFN-γ production and prolonged overall survival, we conclude that IFN-γ may be a good biological marker for predicting clinical efficacy of this treatment. Although this prediction cannot be made prior to α-GalCer/DCs administration, the monitoring of IFN-γ production would still be valuable for patients receiving this immunotherapy. Although none of the cases showed significant tumor regression, the overall MST of all 17 patients (18.6 months) was superior to that of patients with best supportive care (4.6 months) or those treated with other types of therapies (average 10 months) in Figure 1B (37–40).

In the case of the head and neck tumors, we used a combination therapy with α-GalCer/DCs (10^8^) and activated NKT cells (5 × 10^7^) and completed 10 cases, including patients with pharyngeal, laryngeal, esophageal, maxillary, and oral carcinomas, who had advanced or recurrent disease after standard treatments (41). All treated patients showed either a partial response or achieved a stable disease state, indicating significant clinical efficacy (Figure 1C), which was associated with significant NKT cell infiltration into the tumor *in situ* (Figure 1D). To evaluate clinical efficacy, a computed tomography (CT) scan was performed a few days before enrollment and also after the treatment. In some cases with partial responses, we observed that the enhanced area decreased in size, and necrosis appeared at the center of the tumor.

These encouraging clinical studies on advance lung cancers and head and neck tumors warrant further evaluation of NKT cell-targeted immunotherapy for survival benefit. In general, the immunotherapy may be more effective in patients with low tumor burden. Currently, we have been conducting α-GalCer/DC therapy for stage IIA to IIIA lung cancer patients with small tumor foci, including remaining micro-metastasis after radical surgery or after receiving the established first-line therapy in collaboration with National Hospital Organization.

## Future Directions for NKT Cell-Mediated Cancer Therapy Using iPS-Derived NKT Cells

Although an NKT cell-targeted therapy has been shown to have significant clinical efficacy, only one third of patients are eligible in the case of advanced non-small lung cancer patients; the frequency of NKT cells in the other patients is too low. To overcome this problem, we established *in vitro* methods for generation of unlimited numbers of functional NKT cells, which then can be transferred into the patients whose endogenous NKT cell numbers are limited.

Induced pluripotent stem (iPS) cells were generated from mature NKT cells using *Oct3/4, Sox2, Klf4*, and *c-Myc* genes and then were developed into functional NKT cells *in vitro* in the presence of IL-7 and Flt3L according to the conventional protocol (42–44). The NKT cells generated *in vitro* from iPS-NKT cells were functional in the *in vivo* setting using the experimental model of OVA as an artificial tumor antigen (44). When NKT-KO mice were reconstituted with iPS-derived NKT cells followed by immunization with OVA and α-GalCer, we detected a 70-fold increase in the number of OVA-specific IFN-γ producing CD8^+^ T cells above that seen in the control mice (Figure 2A). Under these conditions, the growth of the OVA-expressing EL4 (EG7) tumor cells was suppressed (Figure 2B). Thus, the iPS-derived NKT cells are able to function *in vivo*.

Before any clinical application of iPS-derived NKT cells, two immunological issues need to be addressed, one is whether GvHD is induced by NKT cells and the other is whether semi-allogeneic NKT cells will work *in vivo*, because of the clinical use of iPS-derived NKT cells under semi-allogeneic conditions. To address the first question, iPS-derived NKT cells on a B6 background and B6 or BALB/c CD4 T cells were injected into BALB/c RAG-KO mice. The results were very clear: only B6 CD4T cells, but not iPS-derived B6 NKT cells or BALB/c CD4 T cells, induced GvHD characterized by weight loss, diarrhea, skin disease development, or death after cell transfer. Concerning the second issue of the functional potential of semi-allogeneic NKT cells *in vivo* (129xB6) F1 NKT cells derived from cloned ES cells established by nuclear transfer of mature NKT cells into unfertilized eggs were injected into B6 NKT-KO mice and analyzed for their adjuvant activity in the OVA model. Significant proliferation of OVA-specific CD8 killer T cells was detected, even though these cells are eliminated in a few days. The ability to generate NKT cells using a simple *in vitro* culture system offers a powerful approach for the establishment of optimal NKT cell therapy. Our clinical application of the iPS-derived NKT cell therapy program has now been selected as a Center for Clinical Application Research on Specific Disease/Organ (Type B) in the Research Center Network for Realization of Regenerative Medicine, Japan.

## Future Directions for the Next Generation of NKT Cell-Targeted Therapy

For the establishment of the next generation of NKT cell-targeted therapy, we developed artificial adjuvant vector cells to induce both innate and long-term memory CD8T cell responses against cancer. In this system, allogeneic NIH3T3 fibroblasts were used as a vector cell, into which tumor antigen mRNA and CD1d with α-GalCer were introduced. In the model experiment, we used OVA mRNA as an artificial tumor antigen together with α-GalCer/CD1d to induce the NKT cell-mediated adjuvant effects *in vivo in situ* (Figure 2C) (22). The allogeneic artificial vector cells were destroyed by the host immune system soon after inoculation and all materials carried by the cells were taken up by the host DCs, which immediately stimulated host NKT cells followed by induction of DC maturation and also by activation of innate NK cells and adaptive OVA-specific CD8 killer T cells. Surprisingly, long-term memory CD8 T cell responses were induced in an antigen-specific manner and persisted even 1 year after the initial single injection and suppressed OVA-expressing tumor cell metastasis (Figures 2D,E) (45). To test if this method could be generalized, we used TRP-2, tyrosinase related protein-2, which is a weak tumor antigen expressed by both mouse and human melanoma cells as the tumor antigen, and successfully suppressed tumor growth *in vivo*. Therefore, the artificial vector cells should be useful in the future for vaccines against various tumors.

## Summary

Natural killer T cells bridge innate and adaptive immunity, which enhances protective immune responses and also establishes long-term memory responses. Therefore, NKT cells have important therapeutic potential. In support of this notion, clinical trials on NKT cell-targeted therapy have demonstrated clinical safety and significant clinical efficacy in terms of prolonged median overall survival time in lung cancer patients and achieved stable disease status or partial responses in head or neck cancer patients.

The powerful treatment options for the future are to establish iPS cells that can generate unlimited numbers of NKT cells with adjuvant activity *in vitro* and suppress tumor growth *in vivo*. The other option is to establish the artificial adjuvant vector cells containing tumor mRNA and α-GalCer/CD1d, which have been shown to induce tumor-specific long-term memory CD8T cell responses and to inhibit tumor growth even 1 year after single injection. Thus, these could be therapeutic candidates for the next generation of NKT cell-targeted therapy.

## Conflict of Interest Statement

The authors declare that the research was conducted in the absence of any commercial or financial relationships that could be construed as a potential conflict of interest.
